# RNAPII Degradation Factor Def1 Is Required for Development, Stress Response, and Full Virulence of *Magnaporthe oryzae*

**DOI:** 10.3390/jof9040467

**Published:** 2023-04-13

**Authors:** Xinrong Zhang, Dong Li, Jun Zhu, Jing Zheng, Hongye Li, Qixuan He, Jun Peng, Shen Chen, Xiao-Lin Chen, Weixiang Wang

**Affiliations:** 1 Beijing Key Laboratory of New Technology in Agricultural Application, National Demonstration Center for Experimental Plant Production Education, College of Plant Science and Technology, Beijing University of Agriculture, Beijing 102206, China; 2 State Key Laboratory of Agricultural Microbiology, Provincial Key Laboratory of Plant Pathology of Hubei Province, College of Plant Science and Technology, Huazhong Agricultural University, Wuhan 430070, China; 3 Environment and Plant Protection Institute, Chinese Academy of Tropical Agricultural Sciences, Haikou 571101, China; 4 Guangdong Provincial Key Laboratory of High Technology for Plant Protection, Plant Protection Research Institute, Guangdong Academy of Agricultural Sciences, Guangzhou 510640, China

**Keywords:** *Magnaporthe oryzae*, Def1, pathogenicity, *O*-GlcNAc, stress response, infection

## Abstract

The RNA polymerase II degradation factor Degradation Factor 1 (Def1) is important for DNA damage repair and plays various roles in eukaryotes; however, the biological role in plant pathogenic fungi is still unknown. In this study, we investigated the role of Def1 during the development and infection of the rice blast fungus *Magnaporthe oryzae*. The deletion mutant of *Def1* displayed slower mycelial growth, less conidial production, and abnormal conidial morphology. The appressoria of Δ*def1* was impaired in the penetration into host cells, mainly due to blocking in the utilization of conidial storages, such as glycogen and lipid droplets. The invasive growth of the Δ*def1* mutant was also retarded and accompanied with the accumulation of reactive oxygen species (ROS) inside the host cells. Furthermore, compared with the wild type, Δ*def1* was more sensitive to multiple stresses, such as oxidative stress, high osmotic pressure, and alkaline/acidic pH. Interestingly, we found that Def1 was modified by *O*-GlcNAcylation at Ser232, which was required for the stability of Def1 and its function in pathogenicity. Taken together, the *O*-GlcNAc modified Def1 is required for hyphae growth, conidiation, pathogenicity, and stress response in *M. oryzae*. This study reveals a novel regulatory mechanism of *O*-GlcNAc-mediated Def1 in plant pathogenic fungi.

## 1. Introduction

Environmental factors, such as radiation, chemicals, and even byproducts of cellular metabolism, can cause DNA damage [1]. Affected by DNA damage, transcription may experience severe stalling, pausing, or backtracking, described as transcription stress, with the block of RNA polymerase (RNAP) elongation [2]. Since prolonged stalling of transcription will result in cellular dysfunction, senescence, even cell cycle arrest, and apoptosis, restoring transcription as soon as possible and maintaining gene expression becomes particularly important [3,4]. In fact, the DNA damages in the transcribed strands of active genes are repaired more preferentially than those in non-transcribed regions of the genome; that is, transcription-coupled DNA repair (TCR) takes precedence over global genome DNA repair (GGR) [5,6,7]. TCR is a sub-pathway of nucleotide excision repair (NER) and is evolutionarily conserved from prokaryotes to eukaryotes [8]. The sensor to trigger TCR is the RNAP that blocks the DNA lesions [9,10]. When RNAP encounters a DNA lesion, it remains attached and stays at its template rather than dissociates. This mechanism ensures the high fidelity of long transcripts, but on the other hand, it also blocks all transcription of this gene and occludes the access of nucleotide excision repair factors to the site of disruption; therefore, the RNAP must be cleared away [11]. Most transcription stalled by DNA damage is repaired by TCR. However, if the TCR pathway fails to restore the transcription, an alternative pathway is necessary to remove the stalled RNAP. This alternative pathway is a more drastic “last resort” method, in which the largest subunit of RNAPII, Rpb1, is polyubiquitylated and degraded [12,13,14,15,16,17].

Def1 was discovered 20 years ago in yeast as a protein that forms a complex with Rad26, a helicase involved in TCR [14]. However, Def1 does not take part in TCR; for instead, it is necessary for the degradation of the largest subunit of RNAPII in the “last resort” pathway and was, thus, named the RNAPII Degradation Factor 1 (Def1) [14]. Def1 is largely composed of low-complexity domains, with a coupling of ubiquitin conjugation to ER degradation (CUE) domain in the N-terminal as its only notable feature [18]. The CUE domain, which contains approximately 40 amino acid residues, is moderately conserved and exists in a variety of eukaryotic proteins [19]. It was named after the yeast Cue1p protein, which recruits the ubiquitin-conjugating enzyme to the ER for the degradation of misfolded proteins [20]. The CUE domain, along with UBA (ubiquitin-associated) and UIM (ubiquitin interacting motif) domains, have been well characterized as motifs that bind to monoubiquitin [21,22]. One CUE dimer binds one ubiquitin molecule and then wraps around the ubiquitin [23].

In the “last resort”, Def1 mediates the ubiquitylation and degradation of the subunit of RNAPII via its CUE domain. When DNA damage occurs, Def1 is processed, and its C-terminal domain, which promotes cytoplasmic localization, is removed, allowing the clipped production, pr-Def1, to be transferred from the cytoplasm to the nucleus. In the nucleus, pr-Def1 binds to the monoubiquitylated largest subunit of RNAPII, Rpb1, which was previously ubiquitylated by Rsp5 in a Def1-independent manner. Then pr-Def1 recruits the Elongin-Cullin E3 ligase complex using the CUE domain, forming a stable Rpb1/pr-Def1/Ela1-Elc1 ternary complex. The monoubiquitylated Rpb1 is subsequently polyubiquitylated by the Elongin-Cullin complex and degraded by the proteasomes [2,14,24], resulting in the successful clearance of RNAPII from the DNA lesion. Therefore, Def1 plays a crucial role as in transcription stress.

In recent years, additional functions of Def1 beyond DNA repair have been discovered in yeast, such as transcription promotion [25], synapsis in meiosis [26], oxidative stress response [27], and telomere silencing and maintenance [28,29]. However, the biological role of Def1 in plant pathogenic fungi has not been characterized.

*O*-GlcNAcylation is a type of protein post-translational modification (PTM) where one single N-acetylglucosamine is directly linked to the hydroxyl group of serine or threonine residues through a beta-glycosidic linkage. This modification is found in all metazoans studied thus far [30]. *O*-GlcNAcylation is a reversible and dynamic process that turns over more rapidly than the protein backbone itself [31]. *O*-GlcNAcylation interacts extensively with other PTMs, including phosphorylation, acetylation, methylation, ubiquitination, and proteolysis process [32,33]. *O*-GlcNAc modification acts as a regulator of numerous biological processes, such as nuclear transport, transcription and translation, cell cycle, signal transduction, glucose metabolism, autophagy, and cellular stress response [32,34,35,36,37].

The ascomycete fungus *Magnaporthe oryzae*, which causes a serious threat to rice production, is a model organism for studying plant-pathogen interactions [38,39]. The conidia of *M. oryzae* germinates on the surface of the rice and forms the appressoria with turgor pressure that is sufficient to penetrate the host cuticle using a penetration peg [40]. After intruding into the host cell, it develops invasive hyphae to establish colonization [41]. Some important virulence factors have been identified [42,43]. In this study, we used *M. oryzae* as a model to investigate the roles of Def1 in plant pathogenic fungi. Def1 is involved in multiple functions, including vegetative growth, conidia formation, appressoria development, invasive growth, and stress response. Interestingly, the *O*-GlcNAc modification of Def1 at Ser232 is important for its protein stability and is required for the full virulence of *M. oryzae*. This work highlights the significance of Def1 in plant pathogenic fungi.

## 2. Materials and Methods

### 2.1. Fungal Strains and Growth Conditions

The *M. oryzae* strain P131 was used as a wild type [44]. All strains used in this study, including the wild type, deletion mutants, complementation strains, as well as point-mutation strains (Table S1), were cultured on Oatmeal Tomato Agar (OTA) plates at 28 °C. Colony growth observation and conidiation measurement were performed as described previously [44]. Conidia from 7-day-old colonies cultured on OTA plates were washed down with 0.025% Tween 20 and adjusted to a proper concentration for inoculation. 

### 2.2. Gene Deletion and Complementation

For the deletion of Def1, a gene displacement strategy through split-PCR was used as previously described (Figure S1A) [45]. For protoplast preparation, the wild-type strain was incubated in liquid CM medium, and around 1 g of mycelium was harvested for digestion by Lysing enzyme (Sigma-Aldrich, St. Louis, MO, USA) for 2 h at 150 rpm. The digested protoplast was filtered with three-layer microscope lens papers and washed with 0.7 M NaCl and resuspended with STC buffer (1.2 M Sorbitol, 10 mM Tris [pH 7.5], 50 mM CaCl_2_) and adjusted to a concentration of 1 × 10^8^/mL for transformation. For transformation, the split-PCR products were added into the protoplast (300 μL mixture) and added 2 mLPTC (60% PEG 3350, 10 mM Tris [pH 7.5], 50 mM CaCl_2_) dropwise. The deletion transformants were selected by 250 μg/mL hygromycin B (Roche Diagnostics, Indianapolis, IN, USA) and confirmed by PCR using the Def1 gene-up/gene-down, LCK/HCK-up, RCK/HCK-down primer pairs (Figure S1B; Table S2). For complementation, we inserted the 1.5 kb promoter region and gene-coding region of Def1 into pKN to construct the complementation vector, pKN-Def1, which was transformed into the *Def1* deletion mutant. The complementation transformants were selected by 400 μg/mL neomycin (Amresco, Framingham, MA, USA) and confirmed by PCR using Def1 gene-up/gene-down primer pair (Table S2).

### 2.3. CFW Staining Assay

For observation of the hypha apical cells, mycelia of different strains incubated in liquid complete medium (CM) were harvested and stained with 10 µg/mL Calcofluor White (CFW) for 5 min. For staining of the conidial cells, conidia were harvested from strains incubated on OTA plates for 5 days. The stained hypha and conidia were observed under a fluorescence microscope (Ni90; Nikon, Tokyo, Japan).

### 2.4. Virulence Test and Infection Process Observation

One-month-old rice seedlings (*Oryza sativa* cv. LTH) and one-week-old barley seedlings (*Hordeum vulgare* cv. E9) were used for virulence test. The rice or barley seedlings were sprayed with conidia suspensions (5 × 10^4^ conidia/mL in 0.025% Tween 20) and then incubated with full humidity at 28 °C. The disease lesions were photographed at 5 days post inoculation (dpi). 

For observation of the infection process, conidia suspensions (1 × 10^5^ conidia/mL in 0.025% Tween 20) of different strains were dropped onto the lower barley epidermis, which was then incubated in a dark chamber with full humidity at 28 °C. The infection process was observed at 24 h post-inoculation (hpi) and 30 hpi under a microscope (Ni90; Nikon, Tokyo, Japan).

### 2.5. Glycogen and Lipid Utilization Observation

To observe glycogen and lipid utilization, conidia suspensions (1 × 10^5^ conidia/mL) of different strains was dropped onto a hydrophobic plastic cover glass, which were then stained with staining solution for 10 min at 0, 2, 4, 6, 8, 12, 18, and 24 hpi. KI/ I_2_ solution (60 mg/mL KI, 10 mg/mL I_2_) was used for glycogen staining, and Nile Red solution (50 mM Tris/maleate buffer, 20 mg/mL polyvinylpyrrolidone, 2.5 μg/mL Nile red, pH 7.5) was used for lipid staining. The stained germinating conidia and appressoria were washed with distilled water and photographed under a fluorescence microscope (Ni90; Nikon, Tokyo, Japan).

### 2.6. ROS Accumulation Test

To detect ROS accumulation in host cells infected with *M. oryzae*, conidia suspensions (1 × 10^5^ conidia/mL in 0.025% Tween 20) of different strains were dropped onto the lower barley epidermis. After being incubated in a dark chamber with full humidity at 28 °C for 30 h, the barley leaves were stained with 1 mg/mL 3,3′-diaminobenzidine (DAB) solution (pH 3.8) for 8 h, followed by de-staining with an ethanol/acetic acid solution (ethanol/acetic acid; 94:4) overnight, and then the stained host cells were observed with a microscope (Ni90; Nikon, Tokyo, Japan).

### 2.7. Stress Tolerance Assay

For stress response test, different strains were inoculated onto the CM plates added with 0.1 mg/mL Calcofluor White (CFW), 0.2 mg/mL Congo Red (CR), 0.05% Sodium dodecyl sulfate (SDS), 0.5 M NaCl, 1 M sorbitol, 10 mM H_2_O_2_, or buffered at pH 5.8/pH 6.8/pH 7.8 with phosphate buffer. The colony diameters of different strains were measured at 5 dpi to calculate growth reduction rates [44].

### 2.8. Western Blot

For protein extraction, the mycelia of different strains cultured in liquid CM for 48 h were collected, which were ground into powder in liquid nitrogen and resuspended in protein extraction buffer (Biyuntian, Beijing, China). To detect *O*-GlcNAcylation level, the total protein was immunoprecipitated using anti-Flag beads (Abmart, Shanghai, China), which was then separated on a 10% SDS-PAGE gel and transferred onto a polyvinylidene difluoride (PVDF) membrane (Merck Millipore, Darmstadt, Germany). The PVDF membrane was then incubated with anti-*O*-GlcNAc as the primary antibody (1:5000, Sigma-Aldrich, St. Louis, MO, USA) and anti-rabbit horseradish peroxidase as the secondary antibody (1:10,000, Abmart, Shanghai, China). To detect protein level of Def1, the total protein was separated on a 10% SDS-PAGE gel and, then, transferred onto a PVDF membrane, which was incubated with anti-Flag as the primary antibody (1:5000, Abmart, Shanghai, China) and anti-rabbit horseradish peroxidase as the secondary antibody (1:10,000, Abmart, Shanghai, China).

## 3. Results

### 3.1. Identification of Def1 in M. oryzae

**Figure 1:**
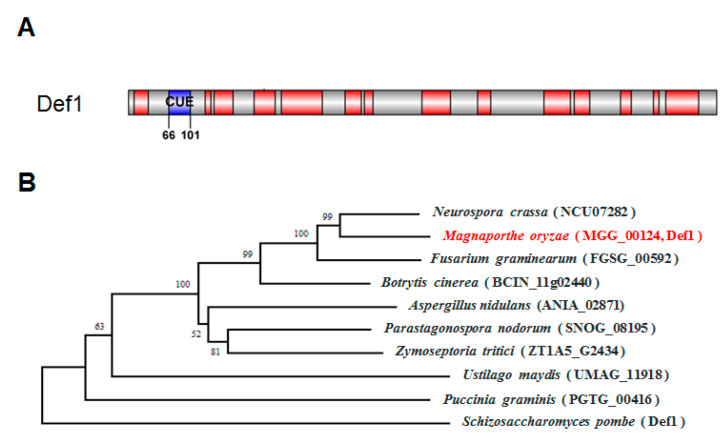
Characterization of Def1 protein in *M. oryzae*. (**A**) Conserved domain of Def1 protein *M. oryzae* predicted by SMART. (**B**) Phylogenetic tree of the Def1 proteins in different organisms using the neighbor-joining method by MEGA software (version 5.2.2).

A single CUE domain-containing protein, MGG_00124, was identified via a search of the *M. oryzae* genome database (Ensembl Fungi, http://fungi.ensembl.org/Magnaporthe_oryzae, accessed on 10 May 2020) by using the *S. cerevisiae* Def1 protein as a query. SMART domain analysis (http://smart.embl-heidelberg.de, accessed on 1 May 2020) showed that the Def1 protein of *M. oryzae* has 951 amino acids with a CUE domain at its N-terminal (Figure 1A). Phylogenetic tree analysis using MEGA suggested the Def1 protein is conserved among fungi, and Def1 in *M. oryzae* is closely related to that in *Neurospora crassa* (NCU07282) and *Fusarium graminearum* (FGSG_00592). Multiple sequence alignment revealed *M. oryzae* Def1 protein shares a 54% positive amino acid identity with that of *N. crassa*, and 52% with *F. graminearum* (Figure 1B). 

Analysis of the whole transcriptome data (authors’ unpublished data) revealed that compared with that in mycelium, the *Def1* gene was highly expressed in conidium, and slightly increased in invasive hyphae formed from 18 to 24 hpi (Figure S2). This result suggested that the expression of the *Def1* gene was fine-tuned during the development and infection process.

To further determine the role of Def1 in *M. oryzae*, we disrupted this gene in the wild-type strain P131 through a gene displacement strategy using a split-marker approach [37]. The transformants were screened by PCR-based methods, and two independent *Def1* deletion mutants, DEF1KO1 and DEF1KO2, which displayed similar phenotypes, were obtained for subsequent analyses (Figure S1). The complementary strains were generated by introducing the native promoter-driven Def1 into the deletion mutant DEF1KO1. As expected, all of the complementary strains showed restored phenotypes, suggesting the phenotypic defects of the deletion mutants resulted from the loss of *Def1*. Then, we selected one complementary strain, cDEF1, for further analysis.

### 3.2. Def1 Contributes to M. oryzae Mycelial Growth and Conidia Formation

**Figure 2:**
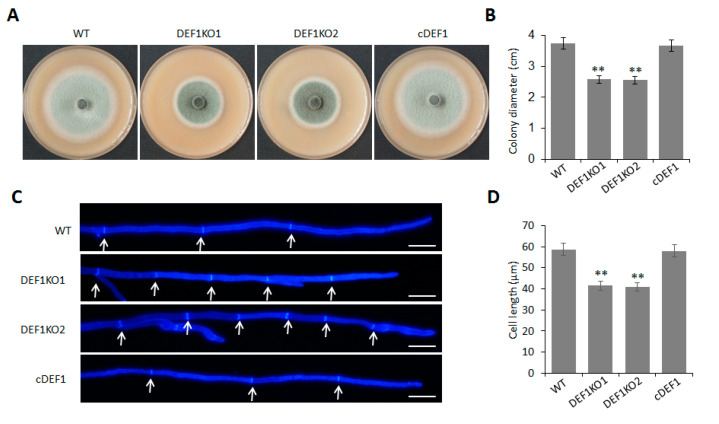
Deletion of *Def1* affects colony growth in *M. oryzae*. (**A**) The colony of different strains grown on OTA plates at 28 °C for 5 days. (**B**) Colony diameters of different strains. Significant differences are labelled with asterisks (**, *p* < 0.01). (**C**) Hypha tips of different strains stained by CFW. Cell septa are indicated with arrows. Bar = 20 μm. (**D**) The length of hypha apical cells of different strains. Significant differences are labelled with asterisks (**, *p* < 0.01). WT, wild type; DEF1KO1 and DEF1KO2, two independent *def1* knock out mutants; cDEF1, complementary strains.

To investigate the role of Def1 in mycelial growth, we observed the colony morphology and size of the Δ*def1*, wild type, and complementary strain cultured on OTA plate at 5 dpi at 28 °C. The colony diameter of Δ*def1* was around 2.6 cm, which was significantly smaller than that of the wild type and complementary strains (~3.8 cm) (Figure 2A,B). Furthermore, Calcofluor White (CFW) staining showed that the average lengths of hypha apical cells of Δ*def1* were significantly shorter than those of the wild type and complementary strains, which accounted for the decreased colony diameter of Δ*def1* (Figure 2C,D). These results indicated that Def1 plays an important role in fungal vegetative growth.

**Figure 3:**
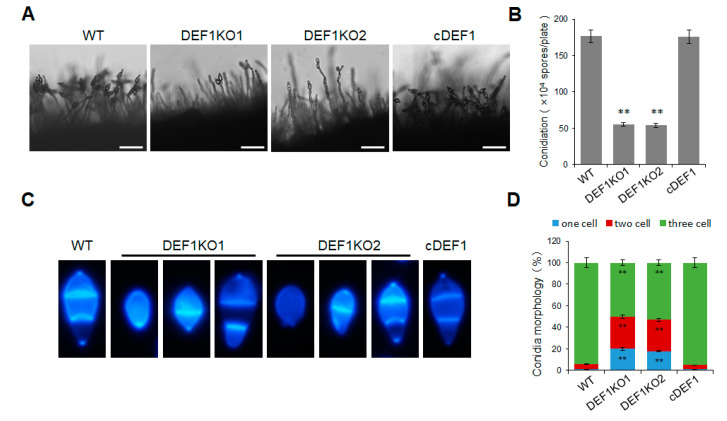
Deletion of *Def1* affects conidium formation in *M. oryzae*. (**A**) Conidiophore of different strains grown on OTA plates were observed under light microscopy. Bar = 50 μm. (**B**) Conidiation of different strains. Conidia on per OTA plates were washed with 30 mL water and counted using a hemocytometer. Significant differences are labelled with asterisks (**, *p* < 0.01). (**C**) Conidia morphology of different strains stained by CFW. (**D**) Percentage of conidia with different septum numbers. For each strain, at least 100 conidia were counted. Significant differences are labelled with asterisks (**, *p* < 0.01). WT, wild type; DEF1KO1 and DEF1KO2, two independent *def1* knock out mutants; cDEF1, complementary strains.

In order to determine whether Def1 is involved in the conidium formation of *M. oryzae*, we observed and quantified the conidia production of the Δ*def1*, wild type and complementary strains. The results showed that the Δ*def1* produced 70% fewer conidia compared to the wild type and complementary strains. Consistent with this, the conidia on the conidiophores in Δ*def1* were much sparser than those in the wild type and complementary strains (Figure 3A,B). In the wild type and complementary strains, more than 90% of the conidia had two septa and three cells, and almost no single-celled conidia were observed. However, in the Δ*def1*, only about 50% of the conidia were three-celled, while nearly 50% were double-celled (one septum) or single-celled (no septum) (Figure 3C,D). This result suggested the conidia in Δ*def1* were obviously abnormal in morphology compared with those in the wild type and complementary strains. Above results suggested that Def1 is crucial for conidium formation.

### 3.3. Def1 Is Required for Full Virulence of M. oryzae

**Figure 4:**
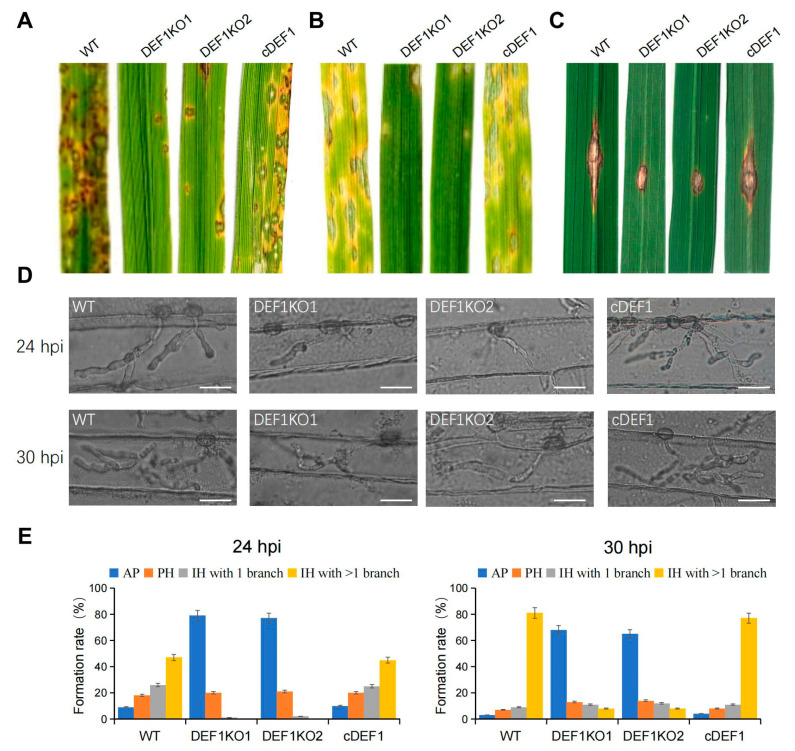
Def1 is required for full virulence of *M. Oryzae*. (**A**,**B**) Conidial suspension (5 × 10^4^ /mL) of different strains were spray onto rice leaves (**A**) and barley leaves (**B**) and incubated for 5 days. (**C**) Mycelia blocks of different strains were inoculated onto the wounds of scratched rice leaves and incubated for 4 days. (**D**) Infection hyphae of different strains in Barley epidermal cells at 24 hpi and 30 hpi. Bar = 20 μm. (**E**) Percentages of appressoria with infection hyphae at different stages. AP, appressorium with no infection hyphae; PH, primary infection hyphae; IH, secondary infection hyphae. WT, wild type; DEF1KO1 and DEF1KO2, two independent *def1* knock out mutants; cDEF1, complementary strains.

To investigate the role of Def1 in pathogenicity, the virulence test was performed by inoculating different strains on susceptible barley and rice plants (*O. sativa* cv. LTH). Conidia suspensions of Δ*def1*, wild type and complementary strains were sprayed on the plant seedlings. Numerous typical lesions appeared on the leaves inoculated with the wild type and complementary strains, while fewer and smaller lesions appeared on the leaves inoculated with Δ*def1* mutants (Figure 4A,B). These findings indicated that Def1 is required for the full virulence of *M. oryzae*.

To determine whether the invasive growth of *M. oryzae* in the host is affected by the deletion of *Def1*, we scratched the rice leaves with a needle, and inoculated mycelia blocks of different strains onto the wounded area of rice leaves. The wild type and complementation strains spread well on the wound rice leaves and formed extended large-sized lesions, but Δ*def1* could not be well extended on the wound rice leaves and formed small-sized lesions (Figure 4C). This result suggested Def1 is important for the colonization and expansion of *M. oryzae* in host cells.

### 3.4. Def1 Is Important for Appressorial Penetration and Invasive Growth

To further reveal why the Δ*def1* lost full virulence, we observed the fungal infection process in barley epidermis cells. We found that the Δ*def1* was obviously blocked in both appressorial penetration and invasive growth during infection. At 24 hpi, more than 90% of the appressoria of the wild type and complementary strains have penetrated into the plant cells, with 75% of them having developed secondary infection hyphae (IH) with branches. In contrast, nearly 80% of appressoria of Δ*def1* could not penetrate into the host cells, and the infection hyphae were mostly without branches. At 30 hpi, more than 80% of the appressoria of the wild type and complementary strains had developed branched secondary infection hyphae, while it was no more than 10% in the Δ*def1* mutants. Moreover, over 60% of the appressoria of Δ*def1* still blocked in penetration (Figure 4D,E). These results indicated that Def1 plays crucial roles in both appressorial penetration and invasive growth of *M. oryzae* during infection.

### 3.5. Def1 Affects Utilization of Glycogen and Lipid during Appressorium Development

**Figure 5:**
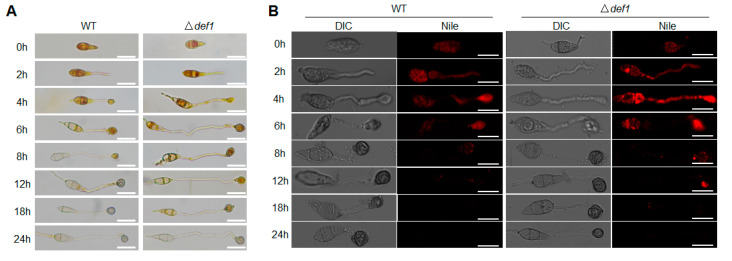
Def1 affects utilization of glycogen and lipid during appressorium development. Stain the wild type (WT) and knock out mutant Δ*def1* with I_2_/KI solution (**A**) and Nile Red (**B**) and observed at different time points during appressorium development. Glycogen was stained and exhibited yellowish-brown color. Lipid was stained and fluoresce red. Bar = 20 μm.

Glycogen and lipid metabolism are essential during the infection process. The utilization efficiency of glycogen and lipid stored in the conidia directly affects the development and function of the appressorium. Since the appressorium formed by Δ*def1* is defective in penetration, we next investigated whether the utilization of glycogen and lipid was blocked. I_2_/KI solution was used to stain the glycogen, and Nile Red was used to stain the lipid during appressorium formation. In the wild type, along with conidium germination and appressorium development, glycogen was completely transferred from the conidium to the appressorium at 8 hpi, and fully utilized at 12 hpi. However, in the Δ*def1* mutant, glycogen could be observed until 18 hpi, and not be completely utilized until 24 hpi (Figure 5A). Similar results were observed for the lipid staining assay. In the wild type, the lipid was transferred from the conidium to the appressorium and was fully utilized at 12 hpi. While in the Δ*def1* mutant, the lipid could not be fully utilized until 18 hpi (Figure 5B). These results indicated that the utilization of glycogen and the lipid in the Δ*def1* mutant was obviously blocked, suggesting that Def1 is involved in glycogen and lipid metabolism, which is of great significance for functional appressorium formation.

### 3.6. Deletion of Def1 Results in Accumulation of Host Reactive Oxygen Species (ROS)

**Figure 6:**
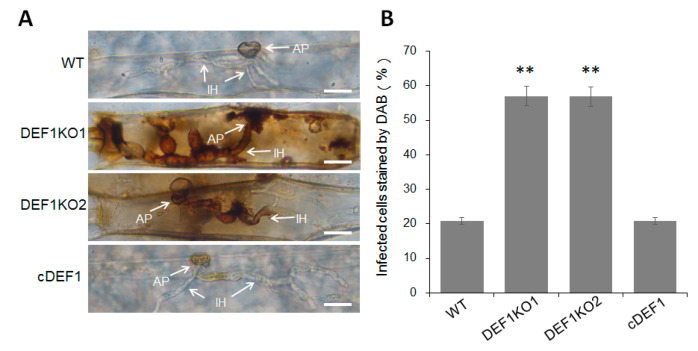
DAB staining assay. (**A**) Barley epidermis cells were inoculated with different strains and then stained with DAB at 30 hpi, and observed after decolorization. Arrows indicate appressoria (AP) and infection hyphae (IH). Bar = 20 μm. (**B**) Percentages of infected cells which stained by DAB. Significant differences are labelled with asterisks (**, *p* < 0.01). WT, wild type; DEF1KO1 and DEF1KO2, two independent *def1* knock out mutants; cDEF1, complementary strains.

Since the invasive growth of Δ*def1* is blocked in host cells, we next investigated whether the invasion of Δ*def1* induced host ROS accumulation. Barley epidermis cells, which inoculated with Δ*def1*, the wild type and complementary strains, were stained with DAB and observed under a microscope after decolorization. Only about 20% of the cells infected by wild type and complementary strains were stained as a brown-reddish color, representing as ROS accumulation. While more than 50% of the cells infected by Δ*def1* were stained and showed a reddish-brown color (Figure 6A,B), suggesting that Def1 plays a role in inhibiting host ROS accumulation during infection.

### 3.7. Def1 Is Involved in Stress Response

**Figure 7:**
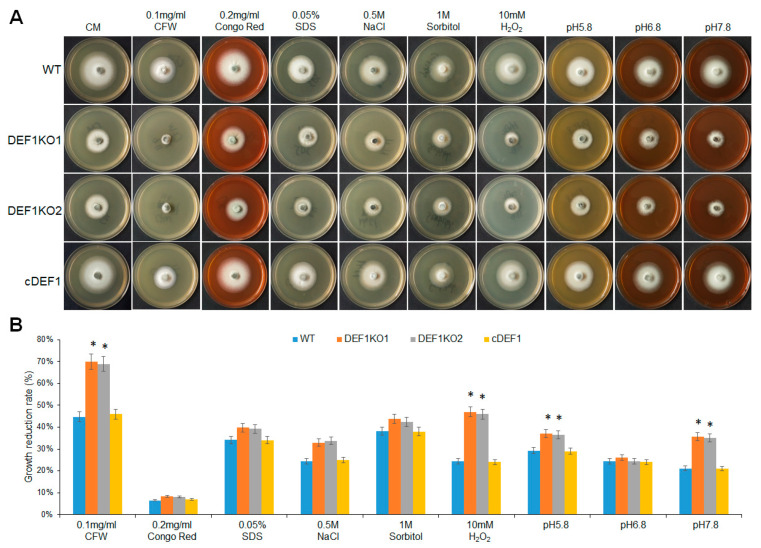
Def1 is involved in various stresses responses. (**A**) The colony morphology of different strains grown on CM plates containing different stress agents at 28 °C for 5 days. (**B**) Growth reduction rates calculated by colony diameter of different strains growing under different stress for 5 days. Significant differences are labelled with asterisks (*, *p* < 0.05). WT, wild type; DEF1KO1 and DEF1KO2, two independent *def1* knock out mutants; cDEF1, complementary strains.

To explore the impact of Def1 disruption on stress response, different strains were subjected to various stress treatments, including cell wall perturbing reagents [Calcofluor white (CFW), Congo red (CR), or sodium dodecyl sulfate (SDS)], high osmotic pressure (0.5 M NaCl and 1 M sorbitol), oxidative stress (H_2_O_2_), or different pH conditions (pH 5.8, pH 6.8 and pH 7.8). After growing under stress for five days, the Δ*def1* mutant was more sensitive to cell wall perturbing reagents CFW, oxidative stress, and alkaline pH compared with that of the wild type and complementary strains. The Δ*def1* mutant also showed higher sensitivity to SDS, high osmotic pressures, and acidic pH (Figure 7). These results indicate that Def1 is involved in responding to various stresses. 

### 3.8. O-GlcNAc Modification Affects the Def1 Stability and Is Required for the Def1 Functions

**Figure 8:**
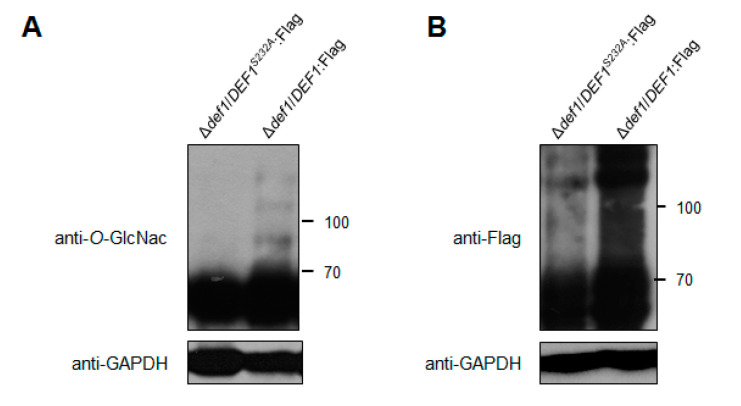
*O*-GlcNAc modification affects the Def1 stability. (**A**) The *O*-GlcNAcylation level of Def1 protein with and without the mutation of *O*-GlcNAc site. Fusion protein DEF1^S232A^:Flag and DEF1:Flag were extracted from Δ*def1* and were immunoprecipitated by anti-Flag Beads, and then blotted with *O*-GlcNAc antibody. (**B**) Protein level of Flag fused Def1 with and without the mutation of *O*-GlcNAc site. Total proteins from extracts of Δ*def1*/*DEF1^S232A^*:Flag and Δ*def1*/DEF1:Flag were detected by anti-Flag antibody.

Through the *M. oryzae O*-GlcNAc proteome data (authors’ unpublished data), we identified Def1 as an *O*-GlcNAcylated protein at site Ser232. To confirm this, we expressed a fusion protein of Def1:Flag in the Δ*def1* strain, with or without substitution at position 232 from Ser to Ala (S232A). The fusion proteins were immunoprecipitated using anti-Flag beads from cell extracts and then detected the *O*-GlcNAcylation of Def1 by an anti-*O*-GlcNAc antibody. As shown in Figure 8A, the *O*-GlcNAcylation level of Def1^S232A^ was significantly decreased, confirming that Def1 was a *O*-GlcNAc modified protein at Ser232.

It has been reported that *O*-GlcNAc modification plays a role in protein stability [46,47]. To investigate whether the functional defects in Δ*def1*/*DEF1^S232A^* result from the decrease of protein stability, we measured the protein level of Def1 with or without the mutation at the *O*-GlcNAc site using an anti-Flag antibody. As shown in Figure 8B, the protein amount of Def1^S232A^ is much lower than the Def1 protein without site mutation. This result suggested that the *O*-GlcNAc modification is required for the functions of Def1, probably through affecting its protein stability.

**Figure 9:**
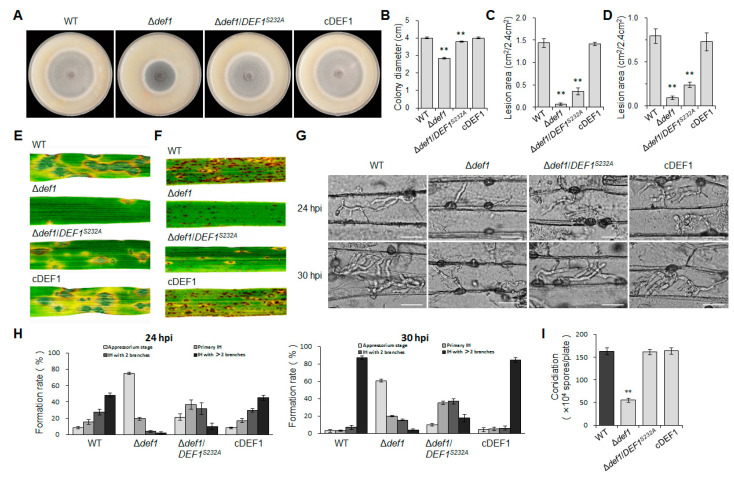
*O*-GlcNAc modification is required for vegetative growth and pathogenicity of *M. oryzae*. (**A**) Colony diameters of different strains. Significant differences are labelled with asterisks (*p* < 0.01). (**B**) Colony morphology of different strains grown on OTA plates at 28 °C for five days. Significant differences are labelled with asterisks (**, *p* < 0.01) (**C**,**E**) Conidial suspension (5 × 10^4^ /mL) of different strains were sprayed onto barley leaves and incubated for 5 days, and then measured the lesion area. Significant differences are labelled with asterisks (**, *p* < 0.01). (**D**,**F**) Conidial suspension (5 × 10^4^ /mL) of different strains were sprayed onto rice leaves and incubated for 5 days, and then measure the lesion area. Significant differences are labelled with asterisks (**, *p* < 0.01). (**G**) Infection hyphae of different strains in Barley epidermal cells at 24 hpi and 30 hpi. Bar = 20 μm. (**H**) Percentages of appressoria with infection hyphae at different stages. AP, appressorium with no infection hyphae; PH, primary infection hyphae; IH, secondary infection hyphae. WT, wild type; Δ*def1*, *def1* knock out mutant; Δ*def1*/*DEF1^S232A^*, *O*-GlcNAc site mutant (Ser232 mutated into Ala); cDEF1, complementary strains. (**I**) Conidiation of different strains. Significant differences are labelled with asterisks (**, *p* < 0.01).

To further confirm whether the *O*-GlcNAc modification has effects on the functions of Def1, we compared the phenotypes of the *O*-GlcNAc site mutant Δ*def1*/*DEF1^S232A^*, Δ*def1*, the wild type, and the complementary strain. The colony diameter of Δ*def1*/*DEF1^S232A^* was slightly smaller than that of the wild type and complementary strains but larger than that of Δ*def1* (Figure 9A,B,), suggesting that the *O*-GlcNAc modification partially participated in vegetative growth. In spraying inoculation experiments performed on barley (Figure 9C,E) and rice (Figure 9D,F) seedlings, both the number and area of lesions caused by Δ*def1*/*DEF1^S232A^* were significantly decreased compared to the wild type and complementary strains, but still slightly increased compared to Δ*def1*. Infection process observation in barley epidermis cells showed that compared to the wild type and complementary strains, the percentage of appressoria in Δ*def1*/*DEF1^S232A^,* which stayed in penetration, was much higher, while the percentage of branched infection hyphae was much lower (Figure 9G,H). These results showed that Δ*def1*/*DEF1^S232A^* was severely blocked in appressorial penetration and invasive growth in a host cell, suggesting that *O*-GlcNAc modification is crucial for Def1 to maintain the virulence of *M. oryzae*. However, the *O*-GlcNAc modification of Def1 does not affect the conidiation (Figure 9I).

## 4. Discussion

In this study, we demonstrated that Def1 plays multiple roles in *M. oryzae*, particularly during the infection process. Our results showed that disruption of Def1 in *M. oryzae* leads to defects in mycelia growth, conidia formation, pathogenicity, and stress response. The Δ*def1* mutants exhibited slower mycelia growth, fewer conidia production, and abnormal conidia morphology. Additionally, during appressoria development, the glycogen and lipid stored in conidia could not be effectively utilized; thus, the appressoria were impaired in penetration to the host cells. Furthermore, when the infection hyphae grew inside the host cell, ROS produced by the host accumulated and blocked the invasive growth and colonization of Δ*def1*. Consequently, the virulence of Δ*def1* decreased seriously. Moreover, we discovered that Def1 was responsible for various stress responses. Finally, we illustrated that Def1 was modified by *O*-GlcNAc at Ser232, which is extremely important for the infection of *M. oryzae*. Our study revealed a novel regulatory mechanism of Def1 through an interesting post-translational modification.

Def1 was originally identified as a protein bound to the TCR factor Rad26 [14]. However, later studies revealed that it is not taking part in TCR but is required for a more drastic “last resort” pathway. In fact, Rad26 blocks Def1 from initiating the Rpb1 degradation to ensure TCR occurs before the last resort [14,48]. The Def1-mediated degradation of Rpb1 not only responds to DNA repair but also responds to conditions that lead to transcription stress [15,49,50,51]. Def1 is necessary for the resistance to multiple DNA damage agents, including those causing double-stranded breaks, bulky lesions, oxidative damage to bases, replication fork collapse, and chromosome rearrangements [14,26,27,52,53,54]. Later, more and more functions of Def1 have been revealed, which extend beyond RNA polymerase degradation. For example, Def1 directly promotes transcription, independent of its Rpb1 degrading activity, but uses its recruitment activity by N-terminal [25]. Def1 also takes part in the silencing and maintenance of telomeres. Telomeres in Δ*def1* cells are shortened by approximately 200 bp and result in a mild silencing defect. Def1 shows genetic interactions with Rrm3 and Pif1, two helicases involved in suppressing DNA damage at telomeric structures, which suggested a role of Def1 in genomic maintenance [28,55]. It is reported that Def1 is required for efficient synapsis between homologues and normal levels of crossover recombination during meiosis [26]. Moreover, Def1 mediates the degradation of excess nucleolar protein to maintain the proteostasis in nucleolus [56]. Although Def1 has a variety of functions in yeast, most of which are centered around maintaining chromosome and genomic integrity [57]. However, the function of Def1 in plant pathogenic fungi had not been investigated previously.

In this study, we found that the impairment of *O*-GlcNAc modification in Def1 leads to a block in appressorial penetration and impairment in invasive growth. It has been reported that *O*-GlcNAcylation modulates protein interaction, stability, and subcellular localization [58], and it can inhibit protein degradation by decreasing their ubiquitination [33,46,47,59]. *O*-GlcNAcylation promotes the binding of deubiquitinase to protect the gluconeogenesis regulator PGC-1a from degradation [46]. Circadian clock relative proteins BMAL1 and CLOCK are rhythmically *O*-GlcNAcylated to stabilize them by inhibiting their ubiquitination [47]. *O*-GlcNAcylation on nucleoporins Nup62 directly reduces its ubiquitylation and proteasomal degradation [60]. In this study, when the *O*-GlcNAc site of Def1 was mutated, the protein level of Def1 was significantly decreased, suggesting that *O*-GlcNAc modification affects the pathogenicity probably through modulating the stability of Def1 protein.

As mentioned previously, Def1 plays a broad role in maintaining chromosome and genomic integrity in yeast. To some extent, this perhaps explains the slower mycelia growth, fewer conidia production, and abnormal conidia with less septa of Δ*def1* in *M. oryzae*. In *M. oryzae*, there is an S-phase cell-cycle checkpoint during appressoria repolarization to organize the penetration to the host cell [61], but whether the defect in penetration of Δ*def1* is resulted from the defective translation stress response in the cell-cycle needs more evidence. As described above, *Δdef1* in *M. oryzae* was sensitive to H_2_O_2_, which can explain the blocking of infection hyphae by ROS in the host cells. Likewise, Δ*def1* in yeast is sensitive to oxidative stress, and Def1 may be important for repairing oxidative DNA damage during transcription [27]. Furthermore, when Def1 is overexpressed, the biosynthesis of glutathione, a major peptide protecting cells against oxidative stress, is activated, indicating the oxidative stress response is increased [62]. Besides oxidative stress, Δ*def1* in *M. oryzae* shows sensitivity to several other stresses, which is consistent with the studies in yeast that 26 different cell-damaging conditions up-regulate the expression of Def1 [63,64,65].

Several reports have revealed that DNA damage repair is important for maintaining the genomic integrity in *M. oryzae*, but to the best of our knowledge, there is no report about the “last resort” pathway. Nijmegen breakage syndrome protein PoNBS1 is involved in DNA repair and development in *P. oryzae*, targeted deletion of PoNBS1 leads to retarded hyphal growth and abnormal conidial germination and shape, but the appressorium formation is normal [66]. The histone acetyltransferase Rtt109 also responds to DNA damage, and the deletion mutant is defective in hyphal growth and asexual reproduction [67]. Transcription factor MoRfx1 regulates the expression of genes involved in cell division and cell wall integrity to affect development and pathogenicity, whose gene’s null mutant displays increased sensitivity to DNA-damaging agents [68].

In summary, our study sheds light on the multiple roles of the RNAPII degradation factor Def1 in plant pathogenic fungi, highlighting its importance in fungal development and pathogenicity.

## Data Availability

Not applicable.

## Supplementary Materials

The following supporting information can be downloaded at: https://www.mdpi.com/article/10.3390/jof9040467/s1. Figure S1: Targeted deletion of *Def1*. (A) Diagram of the deletion strategy of *Def1*. (B) Identification of *Def1* deletion mutants by PCR. KO1 and KO2 are two independent strains. Figure S2: Relative expression level of *Def1* gene in different development stages of *M. oryzae*. HY, Vegetative hyphae; CO, Conidia; AP_3h, Appressoria at 3 hpi; AP_12h, Appressoria at 12 hpi; IH_18h, Infection hyphae at 18 hpi; IH_24h, Infection hyphae at 24 hpi; IH_48h, Infection hyphae at 48 hpi. Table S1. Fungal strains used in this study. Table S2. Primers used in this study.

## References

[1] Ljungman M., Lane D.P. (2004). Transcription—Guarding the genome by sensing DNA damage. Nat. Rev. Cancer.

[2] Wilson M.D., Harreman M., Taschner M., Reid J., Walker J., Erdjument-Bromage H., Tempst P., Svejstrup J.Q. (2013). Proteasome-mediated processing of Def1, a critical step in the cellular response to transcription stress. Cell.

[3] Pani B., Nudler E. (2017). Mechanistic insights into transcription coupled DNA repair. DNA Repair.

[4] Lans H., Hoeijmakers J.H.J., Vermeulen W., Marteijn J.A. (2019). The DNA damage response to transcription stress. Nat. Rev. Mol. Cell Biol..

[5] Bohr V.A., Smith C.A., Okumoto D.S., Hanawalt P.C. (1985). DNA repair in an active gene: Removal of pyrimidine dimers from the DHFR gene of CHO cells is much more efficient than in the genome overall. Cell.

[6] Mellon I., Spivak G., Hanawalt P.C. (1987). Selective removal of transcription-blocking DNA damage from the transcribed strand of the mammalian DHFR gene. Cell.

[7] Mellon I., Hanawalt P.C. (1989). Induction of the *Escherichia coli* lactose operon selectively increases repair of its transcribed DNA strand. Nature.

[8] Hanawalt P.C. (2001). Controlling the efficiency of excision repair. Mutat. Res..

[9] Svejstrup J.Q. (2002). Mechanisms of transcription-coupled DNA repair. Nat. Rev. Mol. Cell Biol..

[10] Hanawalt P.C., Spivak G. (2008). Transcription-coupled DNA repair: Two decades of progress and surprises. Nat. Rev. Mol. Cell Biol..

[11] Donahue B.A., Yin S., Taylor J.S., Reines D., Hanawalt P.C. (1994). Transcript cleavage by RNA polymerase II arrested by a cyclobutane pyrimidine dimer in the DNA template. Proc. Natl. Acad. Sci. USA.

[12] Wilson M.D., Harreman M., Svejstrup J.Q. (2013). Ubiquitylation and degradation of elongating RNA polymerase II: The last resort. Biochim. Biophys. Acta.

[13] Ratner J.N., Balasubramanian B., Corden J., Warren S.L., Bregman D.B. (1998). Ultraviolet radiation-induced ubiquitination and proteasomal degradation of the large subunit of RNA polymerase II. Implications for transcription-coupled DNA repair. J. Biol. Chem..

[14] Woudstra E.C., Gilbert C., Fellows J., Jansen L., Brouwer J., Erdjument-Bromage H., Tempst P., Svejstrup J.Q. (2002). A Rad26-Def1 complex coordinates repair and RNA pol II proteolysis in response to DNA damage. Nature.

[15] Anindya R., Aygun O., Svejstrup J.Q. (2007). Damage-induced ubiquitylation of human RNA polymerase II by the ubiquitin ligase Nedd4, but not Cockayne syndrome proteins or BRCA1. Mol. Cell..

[16] Lommel L., Bucheli M.E., Sweder K.S. (2000). Transcription-coupled repair in yeast is independent from ubiquitylation of RNA pol II: Implications for Cockayne’s syndrome. Proc. Natl. Acad. Sci. USA.

[17] Chen X., Ruggiero C., Li S. (2007). Yeast Rpb9 plays an important role in ubiquitylation and degradation of Rpb1 in response to UV-induced DNA damage. Mol. Cell. Biol..

[18] Ponting C.P. (2002). Novel domains and orthologues of eukaryotic transcription elongation factors. Nucleic Acids Res..

[19] Ponting C.P. (2000). Proteins of the endoplasmic-reticulum-associated degradation pathway: Domain detection and function prediction. Biochem. J..

[20] Biederer T., Volkwein C., Sommer T. (1997). Role of Cue1p in ubiquitination and degradation at the ER surface. Science.

[21] Kang R.S., Daniels C.M., Francis S.A., Shih S.C., Salerno W.J., Hicke L., Radhakrishnan I. (2003). Solution structure of a CUE-ubiquitin complex reveals a conserved mode of ubiquitin binding. Cell.

[22] Buchberger A. (2002). From UBA to UBX: New words in the ubiquitin vocabulary. Trends Cell Biol..

[23] Prag G., Misra S., Jones E.A., Ghirlando R., Davies B.A., Horazdovsky B.F., Hurley J.H. (2003). Mechanism of ubiquitin recognition by the CUE domain of Vps9p. Cell.

[24] Harreman M., Taschner M., Sigurdsson S., Anindya R., Reid J., Somesh B., Kong S.E., Banks C.A., Conaway R.C., Conaway J.W. (2009). Distinct ubiquitin ligases act sequentially for RNA polymerase II polyubiquitylation. Proc. Natl. Acad. Sci. USA.

[25] Damodaren N., Van Eeuwen T., Zamel J., Lin-Shiao E., Kalisman N., Murakami K. (2017). Def1 interacts with TFIIH and modulates RNA polymerase II transcription. Proc. Natl. Acad. Sci. USA.

[26] Jordan P.W., Klein F., Leach D.R. (2007). Novel roles for selected genes in meiotic DNA processing. PLoS Genet..

[27] Wang P., Byrum S., Fowler F.C., Pa S., Tackett A.J., Tyler J.K. (2017). Proteomic identification of histone post-translational modifications and proteins enriched at a DNA double-strand break. Nucleic Acids Res..

[28] Chen Y.B., Yang C.P., Li R.X., Zeng R., Zhou J.Q. (2005). Def1p is involved in telomere maintenance in budding yeast. J. Biol. Chem..

[29] Makovets S., Herskowitz I., Blackburn E.H. (2004). Anatomy and dynamics of DNA replication fork movement in yeast telomeric regions. Mol. Cell Biol..

[30] Hart G.W., Housley M.P., Slawson C. (2007). Cycling of O-linked beta-*N*-acetylglucosamine on nucleocytoplasmic proteins. Nature.

[31] Roquemore E.P., Chevrier M.R., Cotter R.J., Hart G.W. (1996). Dynamic O-GlcNAcylation of the small heat shock protein alpha B-crystallin. Biochemistry.

[32] Vercoutter-Edouart A.S., El Yazidi-Belkoura I., Guinez C., Baldini S., Leturcq M., Mortuaire M., Mir A.M., Steenackers A., Dehennaut V., Pierce A. (2015). Detection and identification of O-GlcNAcylated proteins by proteomic approaches. Proteomics.

[33] Yang X., Qian K. (2017). Protein O-GlcNAcylation: Emerging mechanisms and functions. Nat. Rev. Mol. Cell Biol..

[34] Chatham J.C., Marchase R.B. (2010). Protein O-GlcNAcylation: A critical regulator of the cellular response to stress. Curr. Signal Transduct. Ther..

[35] Han C., Gu Y., Shan H., Mi W., Sun J., Shi M., Zhang X., Lu X., Han F., Gong Q. (2017). O-GlcNAcylation of SIRT1 enhances its deacetylase activity and promotes cytoprotection under stress. Nat. Commun..

[36] Guo B., Liang Q., Li L., Hu Z., Wu F., Zhang P., Ma Y., Zhao B., Kovacs A.L., Zhang Z. (2014). O-GlcNAc-modification of SNAP-29 regulates autophagosome maturation. Nat Cell Biol..

[37] Ruba A., Yang W. (2016). O-GlcNAcylation in the nuclear pore complex. Cell Mol. Bioeng..

[38] Wilson R.A., Talbot N.J. (2009). Under pressure: Investigating the biology of plant infection by *Magnaporthe oryzae*. Nat. Rev. Microbiol..

[39] Yan X., Talbot N.J. (2016). Investigating the cell biology of plant infection by the rice blast fungus *Magnaporthe oryzae*. Curr. Opin. Microbiol..

[40] deJong J.C., McCormack B.J., Smirnoff N., Talbot N.J. (1997). Glycerol generates turgor in rice blast. Nature.

[41] Kankanala P., Czymmek K., Valent B. (2007). Roles for rice membrane dynamics and plasmodesmata during biotrophic invasion by the blast fungus. Plant Cell..

[42] Dixon K.P., Xu J.R., Smirnoff N., Talbot N.J. (1999). Independent signaling pathways regulate cellular turgor during hyperosmotic stress and appressorium-mediated plant infection by *Magnaporthe grisea*. Plant Cell.

[43] Reddy B., Kumar A., Mehta S., Sheoran N., Chinnusamy V., Prakash G. (2021). Hybrid de novo genome-reassembly reveals new insights on pathways and pathogenicity determinants in rice blast pathogen *Magnaporthe oryzae* RMg_Dl. Sci. Rep..

[44] Bansal S., Mallikarjuna M.G., Reddy B., Balamurugan A., Achary V.M.M., Reddy M.K., Kumar A., Prakash G. (2023). Characterization and validation of hypothetical virulence factors in recently sequenced genomes of Magnaporthe species. Physiol. Mol. Plant Pathol..

[45] Goswami R.S. (2012). Targeted gene replacement in fungi using a split-marker approach. Methods Mol. Biol..

[46] Ruan H.B., Han X., Li M.D., Singh J.P., Qian K., Azarhoush S., Zhao L., Bennett A.M., Samuel V.T., Wu J. (2012). O-GlcNAc transferase/host cell factor C1 complex regulates gluconeogenesis by modulating PGC-1α stability. Cell Metab..

[47] Li M.D., Ruan H.B., Hughes M.E., Lee J.S., Singh J.P., Jones S.P., Nitabach M.N., Yang X. (2013). O-GlcNAc signaling entrains the circadian clock by inhibiting BMAL1/CLOCK ubiquitination. Cell Metab..

[48] Xu J., Wang W., Xu L., Chen J.Y., Chong J., Oh J., Leschziner A.E., Fu X.D., Wang D. (2020). Cockayne syndrome B protein acts as an ATP-dependent processivity factor that helps RNA polymerase II overcome nucleosome barriers. Proc. Natl. Acad. Sci. USA.

[49] Somesh B.P., Reid J., Liu W.F., Sogaard T.M., Erdjument-Bromage H., Tempst P., Svejstrup J.Q. (2005). Multiple mechanisms confining RNA polymerase II ubiquitylation to polymerases undergoing transcriptional arrest. Cell.

[50] Sigurdsson S., Dirac-Svejstrup A.B., Svejstrup J.Q. (2010). Evidence that transcript cleavage is essential for RNA polymerase II transcription and cell viability. Mol. Cell..

[51] Hobson D.J., Wei W., Steinmetz L.M., Svejstrup J.Q. (2012). RNA polymerase II collision interrupts convergent transcription. Mol. Cell..

[52] Owiti N., Lopez C., Singh S., Stephenson A., Kim N. (2017). Def1 and Dst1 play distinct roles in repair of AP lesions in highly transcribed genomic regions. DNA Repair.

[53] Stepchenkova E.I., Shiriaeva A.A., Pavlov Y.I. (2018). Deletion of the DEF1 gene does not confer UV-immutability but frequently leads to self-diploidization in yeast *Saccharomyces cerevisiae*. DNA Repair.

[54] Gaillard H., Tous C., Botet J., Gonzalez-Aguilera C., Quintero M.J., Viladevall L., Garcia-Rubio M.L., Rodriguez-Gil A., Marin A., Arino J. (2009). Genome-wide analysis of factors affecting transcription elongation and DNA repair: A new role for PAF and Ccr4-not in transcription-coupled repair. PLoS Genet..

[55] Paeschke K., Bochman M.L., Garcia P.D., Cejka P., Friedman K.L., Kowalczykowski S.C., Zakian V.A. (2013). Pif1 family helicases suppress genome instability at G-quadruplex motifs. Nature.

[56] Morshed S., Mochida T., Shibata R., Ito K., Mostofa M.G., Rahman M.A., Ushimaru T. (2019). Def1 mediates the degradation of excess nucleolar protein Nop1 in budding yeast. Biochem. Biophys. Res. Commun..

[57] Akinniyi O.T., Reese J.C. (2021). DEF1: Much more than an RNA polymerase degradation factor. DNA Repair.

[58] Slawson C., Copeland R.J., Hart G.W. (2010). O-GIcNAc signaling: A metabolic link between diabetes and cancer?. Trends Biochem. Sci..

[59] Ruan H.B., Nie Y.Z., Yang X.Y. (2013). Regulation of protein degradation by O-GlcNAcylation: Crosstalk with ubiquitination. Mol. Cell. Proteom..

[60] Zhu Y., Liu T.W., Madden Z., Yuzwa S.A., Murray K., Cecioni S., Zachara N., Vocadlo D.J. (2016). Post-translational O-GlcNAcylation is essential for nuclear pore integrity and maintenance of the pore selectivity filter. J. Mol. Cell. Biol..

[61] Oses-Ruiz M., Sakulkoo W., Littlejohn G.R., Martin-Urdiroz M., Talbot N.J. (2017). Two independent S-phase checkpoints regulate appressorium-mediated plant infection by the rice blast fungus *Magnaporthe oryzae*. Proc. Natl. Acad. Sci. USA.

[62] Suzuki T., Yokoyama A., Tsuji T., Ikeshima E., Nakashima K., Ikushima S., Kobayashi C., Yoshida S. (2011). Identification and characterization of genes involved in glutathione production in yeast. J. Biosci. Bioeng..

[63] Jelinsky S.A., Estep P., Church G.M., Samson L.D. (2000). Regulatory networks revealed by transcriptional profiling of damaged Saccharomyces cerevisiae cells: Rpn4 links base excision repair with proteasomes. Mol. Cell. Biol..

[64] MacIsaac K.D., Wang T., Gordon D.B., Gifford D.K., Stormo G.D., Fraenkel E. (2006). An improved map of conserved regulatory sites for *Saccharomyces cerevisiae*. BMC Bioinform..

[65] Venters B.J., Wachi S., Mavrich T.N., Andersen B.E., Jena P., Sinnamon A.J., Jain P., Rolleri N.S., Jiang C., Hemeryck-Walsh C. (2011). A comprehensive genomic binding map of gene and chromatin regulatory proteins in *Saccharomyces*. Mol. Cell..

[66] Narukawa-Nara M., Sasaki K., Ishii A., Baba K., Amano K., Kuroki M., Saitoh K.-I., Kamakura T. (2015). Identification and characterization of a novel gene encoding the NBS1 protein in *Pyricularia oryzae*. Biosci. Biotechnol. Biochem..

[67] Kwon S., Lee J., Jeon J., Kim S., Park S.-Y., Jeon J., Lee Y.-H. (2018). Role of the histone acetyltransferase Rtt109 in development and pathogenicity of the rice blast fungus. Mol. Plant Microbe Interact..

[68] Sun D., Cao H., Shi Y., Huang P., Dong B., Liu X., Lin F., Lu J. (2017). The regulatory factor X protein MoRfx1 is required for development and pathogenicity in the rice blast fungus *Magnaporthe oryzae*. Mol. Plant Pathol..

