# Supplementary material for: RNAPII Degradation Factor Def1 Is Required for Development, Stress Response, and Full Virulence of Magnaporthe oryzae
Source: J Fungi (Basel). 2023 Apr 13;9(4):467. doi: 10.3390/jof9040467 (PMC10145571; doi:10.3390/jof9040467)
Supplement: Supplementary file 1 [file jof-09-00467-s001.zip › jof-2254551-supplementary.pdf]

Journal of *Fungi*

MDPI

Article

RNAPII Degradation Factor Def1 Is Required for Development, Stress Response, and Full Virulence of *Magnaporthe oryzae*

Xinrong Zhang [1,2], Dong Li [1], Jun Zhu [2], Jing Zheng [2], Hongye Li [2], Qixuan He [1], Jun Peng [3], Shen Chen [4], Xiao-Lin Chen [2,*] and Weixiang Wang [1,*]

[1] Beijing Key Laboratory of New Technology in Agricultural Application, National Demonstration Center for Experimental Plant Production Education, College of Plant Science and Technology, Beijing University of Agriculture, Beijing 102206, China
[2] State Key Laboratory of Agricultural Microbiology, Provincial Key Laboratory of Plant Pathology of Hubei Province, College of Plant Science and Technology, Huazhong Agricultural University, Wuhan 430070, China
[3] Environment and Plant Protection Institute, Chinese Academy of Tropical Agricultural Sciences, Haikou 571101, China
[4] Guangdong Provincial Key Laboratory of High Technology for Plant Protection, Plant Protection Research Institute, Guangdong Academy of Agricultural Sciences, Guangzhou 510640, China
* Correspondence: chenxiaolin@mail.hzau.edu.cn (X.-L.C.); wangweixiang@bua.edu.cn (W.W.)

Citation: Zhang, X.; Li, D.; Zhu, J.; Zheng, J.; Li, H.; He, Q.; Peng, J.; Chen, S.; Chen, X-L.; Wang, W. RNAPII Degradation Factor Def1 is Required for Development, Stress Response, and Full Virulence of *Magnaporthe oryzae*. *J. Fungi* **2023**, *9*, 467. https://doi.org/10.3390/jof9040467

Academic Editor: Ulrich Kück

Received: 15 February 2023
Revised: 6 April 2023
Accepted: 11 April 2023
Published: 13 April 2023

Copyright: © 2023 by the authors. Submitted for possible open access publication under the terms and conditions of the Creative Commons Attribution (CC BY) license (https://creativecommons.org/licenses/by/4.0/).

Supplementary material – All relevant material referred to, but not included in the main text are included in the following supplementary section. All experimental raw data is available upon request from Prof Xiao-Lin Chen at the College of Plant Science & Technology, Huazhong Agricultural University.

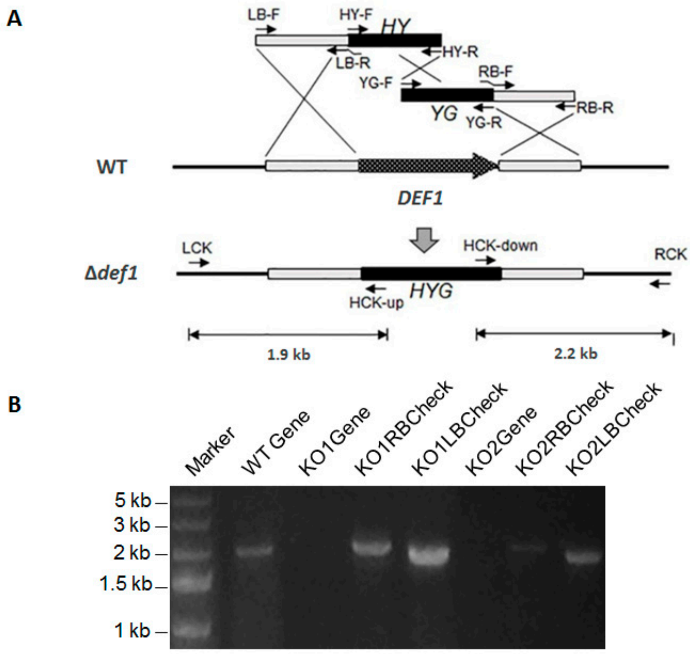

Figure S1. Targeted deletion of *Def1*. (A) Diagram of the deletion strategy of *Def1*. (B) Identification of *Def1* deletion mutants by PCR. KO1 and KO2 are two independent strains.

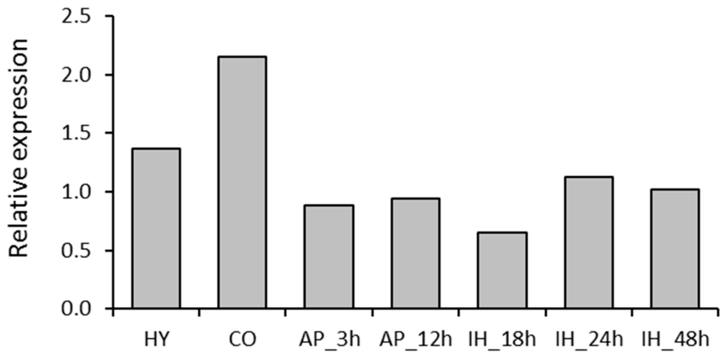

Figure S2. Relative expression level of *Def1* gene in different development stages of *M. oryzae*. HY, Vegetative hyphae; CO, Conidia; AP_3h, Appressoria at 3 hpi; AP_12h, Appressoria at 12 hpi; IH_18h, Infection hyphae at 18 hpi; IH_24h, Infection hyphae at 24 hpi; IH_48h, Infection hyphae at 48 hpi.

Table S1. Fungal strains used in this study.

Strains	Discriptions	References
WT	P131, a wild-type isolate of *M. oryzae*	This study
DEF1KO1	One independent *DEF1* deletion mutant of P131	This study
DEF1KO2	Another independent *DEF1* deletion mutant of P131	This study
cDEF1	Complementary strain of DEF1KO1	This study
Δ*def1*/*DEF1*^{S232A}:Flag	*O*-GlcNAc site mutant strains of DEF1	This study
Δ*def1*/DEF1:Flag	DEF1-Flag fusion expression strains	This study

Table S2. Primers used in this study.

Primers	Sequences (5'–3')
gene-up	CCGAGACTATCGAGCCACTC
gene-down	CACCAAAACCCTGGTTACCA
LCK	TGCCGGTCTCTTTGATACAT
HCK-up	GACAGACGTCGCGGTGAGTT
HCK-down	TCTGGACCGATGGCTGTGTAG
RCK	CGAAGTCGGGTGTAGGCTCT
pKN-F	GGCGAATTGGGTACCAGTTGTTGCGAGTCTTGAGG
pKN-R	GTGGATCCCGAATTCGGCCTAATGGTAGTTGTTGC
pKNFlag-F	AATCTTCAAACCCGGGATGTCTACTGAGGCACAAACCCG
pKNFlag-R	ACGGTATCGATAAGCTTATGGTAGTTGTTGCCCCAAC
DEF1-S232AF	GCTCCTGCTCCTGCCAAACCCGCCGTC
DEF1-S232AR	GACGGCGGGTTTGGCAGGAGCAGGAGC
